# Prenatal Diagnosis and Neurodevelopmental Outcome of Children With Marked Opening of the Fourth Ventricle: Challenges and Pitfalls in MRI Diagnostic Criteria

**DOI:** 10.1002/pd.70093

**Published:** 2026-02-20

**Authors:** Léa Schieffer, Catherine Garel, Laurent Guibaud, Christelle Rougeot‐Jung, Lydie Burglen, Mona Massoud, Dorothée Ville, Eléonore Blondiaux, Jean Marie Jouannic, Sara Cabet, Vincent DesPortes, Stéphanie Valence

**Affiliations:** ^1^ Service de Neuropédiatrie Hospices Civils de Lyon Lyon France; ^2^ Service de radiologie Hôpital Armand‐Trousseau Paris France; ^3^ Service de radiologie Hospices Civils de Lyon Lyon France; ^4^ Servie de génétique Hôpital Armand‐Trousseau Paris France; ^5^ Service d'obstétrique Hospices Civils de Lyon Lyon France; ^6^ Service d'obstétrique Hôpital Armand‐Trousseau Paris France; ^7^ Service de neuropédiatrie Hôpital Armand‐Trousseau Paris France

## Abstract

**Introduction:**

The neurodevelopmental outcome of ‘Cystic’ malformations of the posterior fossa with marked opening of the fourth ventricle, such as Dandy Walker malformation (DWM) and large Blake's pouch cyst (BPC), is a major issue. This study aimed to refine relevant MRI criteria for distinguishing DWM from BPC and identify prognostic factors.

**Patients and Methods:**

Inclusion criteria were prenatal retrocerebellar fluid space diameter > 10 mm, marked opening of the fourth ventricle with a tegmento‐vermian angle (TVA) > 40°, and postnatal follow‐up > 2 years.

**Results:**

27 patients were classified as follows: 6 DWM characterized by an overall upward orientation of the tentorium, an open tegmento‐tentorial angle (TTA > 78 ) and a high TVA (median 132°); 15 BPC with a normal downward orientation of the proximal part of the tentorium (TTA < 68°) and distal upward displacement (median TVA 74°); 3 PHACE syndromes (Posterior fossa abnormalities, Haemangioma, Arterial cerebrovascular anomalies, Cardiac defects, Eye anomalies) and 3 unclassified. Four prognostic factors were identified, (i) diagnosis: DWM (two deaths, three learning disabilities and one typical development (TD)) versus BPC (five learning disabilities [4/5 with associated malformation or genetic defects] and 10 TD); (ii) associated versus isolated (36% vs. 87% TD); (iii) obstructive ventriculomegaly versus no hydraulic complications (20% vs. 91% TD); and (iv) the foetal TVA value and clinical outcome (correlation coefficient = 0.561, *p* = 0.006).

## Summary

- What is already known about this topic?
  ◦ Neurodevelopmental outcome of ‘cystic’ malformations of the posterior fossa with markedly opening of the fourth ventricle is a major issue due to their wide variability
  ◦ The relevant MRI criteria for distinguishing Dandy‐Walker malformation (DWM) from a large Blake pouch cyst (BPC) need to be refined
- What does this study add?
  ◦ The tegmento‐tentorial angle is relevant for distinguishing DWM from large BPC
  ◦ The foetal tegmento‐vermian angle appears to be a prognostic marker to consider

## 1 Introduction

The management of posterior fossa malformations with enlarged retrocerebellar fluid space, usually called ‘cystic’ malformations of the posterior fossa (CMPF) and detected prenatally, remains challenging since some are associated with cognitive impairment [1, 2]. Among those with an opening of the fourth ventricle (V4), four entities have been described: Dandy Walker malformation (DWM), persistent Blake's pouch cyst (BPC), PHACE syndromes (Posterior fossa abnormalities, Haemangioma, Arterial cerebrovascular anomalies, Cardiac defects, Eye anomalies) and rare cases of expansive porencephalic cyst [3]. DWM is classically defined by elevation of the tentorium, opening of the V4 and vermian dysgenesis [3, 4, 5, 6]. Studies of the neurological outcome of DWM are scarce, with small cohorts and short follow‐up [7]. The prognosis is variable; however, it is more severe when associated with other anomalies [7, 8, 9, 10]. BPC is defined by a retrocerebellar cyst with opening of the V4^3, 7^. The vermis is presumed to be intact. From an embryological point of view, the two entities (DWM and BPC) are reported to have different origins [3]. BPC is described as having a more favourable neurological prognosis than DWM [7]. Thus, prenatal counselling is currently driven by differential diagnoses of either BPC (‘favourable’ prognosis) or DWM (‘uncertain’ prognosis).

Foetal ultrasound and MRI are used to distinguish between these different entities [3]. Vermian height (diminished in DWM), vermis morphology (dysgenesis in DWM) and the tegmento‐vermian angle (TVA), which measures the opening of the V4, are classic criteria to differentiate between DWM and BPC [11, 12]. However, in our experience, some large BPCs confirmed on post‐natal imaging showed a mass effect on the vermis, complicating the assessment of their morphology and size [11].

**FIGURE 1:**
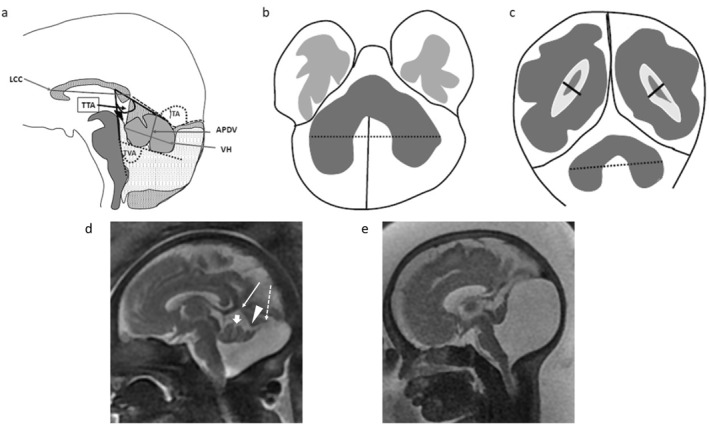
Schematic diagram of measurements taken. (a) Midsagittal slice. TVA: tegmento‐vermian angle; ITA: intra‐tentorial angle; TTA: tegmento‐tentorial angle; LCC: length of the corpus callosum; VH: vermian height; APDV: anteroposterior diameter of the vermis. (b) Axial slice at the level of the cerebellar hemispheres. The dotted line indicates transverse cerebellar diameter (TCD). The anteroposterior diameter of the retrocerebellar fluid space is represented by a plain line. (c) Coronal slice at the level of the atria indicating ventricular diameter (plain lines) and transverse cerebellar diameter (TCD) (dotted line). (d–e) Prenatal MRI with T2‐weighted midsagittal slices showing the typical appearance of Blake's pouch and Dandy‐Walker malformation: (d) Patient TRS9; 31 + 4 weeks of gestation with Blake's pouch. Notice the typical downward orientation of the proximal part of the tentorium (plain arrow) with a sharp tegmento‐tentorial (TTA) angle. The tentorium shows elevation of the distal part (dotted arrow) leading to an obvious intra‐tentorial (ITA) angle (arrowhead). The vermian main axis is tilted upwards, resulting in a moderately increased tegmento‐vermian angle. Vermian foliation is visible (primary fissure: thick arrow). (e) Patient TRS14; 32 + 1 week of gestation with Dandy‐Walker malformation. Notice the global upward orientation of the tentorium including the proximal part, leading to a markedly increased tegmento‐tentorial angle. No intra‐tentorial angle is visible. The vermian main axis is markedly tilted upwards, resulting in a markedly increased tegmento‐vermian angle. Vermian foliation is not visible and the anteroposterior diameter of the vermis is reduced in size.

We previously proposed features that distinguish a DWM from a large BPC based on the orientation of the tentorium: DWM are characterized by an overall upward orientation of the tentorium with an open tegmento‐tentorial angle (TTA), without any detectable intra‐tentorial change (no intra‐tentorial angle [ITA]); conversely, large BPC is defined by a normal downward orientation of the proximal part of the tentorium (sharp TTA), with a change of direction of the distal part of the tentorium leading to an obvious ITA [3] (Figure 1a).

This study aimed to refine the relevant foetal and postnatal MRI criteria that may be used to distinguish between DWM and large BPC, assess the developmental outcome of these two groups of patients, and identify imaging criteria and/or clinical features relevant as prognostic factors.

## 2 Patients and Methods

### 2.1 Patients

We carried out a two‐centre retrospective study on children born to pregnant women referred to tertiary units due to a CMPF, between 01/01/2006 and 31/12/2021. All patients included in the study met the following three inclusion criteria: (i) prenatal diagnosis of enlarged retrocerebellar fluid space (anteroposterior diameter > 10 mm) with marked opening of the V4, characterized by a TVA > 40°; (ii) available foetal and postnatal brain MRI; and (iii) postnatal follow‐up of at least two years.

### 2.2 Imaging Data

Foetal and postnatal brain MRI were performed using 1.5 T (Philips, Ingenia, Amsterdam, Holland and GE Healthcare, Optima, Chicago, USA), with T2‐weighted sequences in all three planes of space. Images were viewed on Centricity Universal Viewer (GE Healthcare, Chicago, USA) in Lyon and Carestream vue PACS (Rochester, USA) in Paris.

The following measures were recorded (Figure 1a–c; value and percentile according to gestational or postnatal age) [13, 14]: anteroposterior diameter of the vermis, height of the vermis (craniocaudal diameter), transverse diameter of the cerebellum (coronal plane), anteroposterior diameter of the retrocerebellar space (axial plane), vermian morphology (subjective evaluation with complete/incomplete appearance in midsagittal plane), morphology and symmetry of the cerebellar hemispheres (in axial plane), and shape of the brainstem. Three angles were measured on a median sagittal plane (Figure 1a): (i) the TVA between the posterior aspect of the brainstem and the tangent to the anterior aspect of the vermis, corresponding to the opening angle of the V4; (ii) the TTA between the posterior aspect of the brainstem and the cerebellar tentorium; and (iii) the ITA reflecting a change of direction of the tentorium. Associated abnormalities were also assessed (Figure 1b–c): morphology and length of the corpus callosum (midsagittal plane), heterotopia/cortical abnormalities, and the presence of ventriculomegaly (> 10 mm; on coronal plane).

As mentioned in the introduction, the diagnoses of DWM and large BPC were indicated on the basis of tentorium orientation according to the angles of TTA and ITA [3] (Figure 1d–e). PHACE syndrome was indicated when a small unilateral cerebellar hemisphere with the “tilted telephone receiver” appearance was observed [15].

Five important methodological criteria were applied: (i) MRI was interpreted separately by two paediatric neuroradiologists (LG and CG); (ii) if diagnostic criteria could not be validated by both radiologists, the patient was considered “unclassified”; (iii) foetal MRI was initially read without prior knowledge of the post‐natal MRI diagnosis or patient outcome; (iv) patients were classified according to postnatal MRI; and (v) concordance between prenatal imaging diagnosis and final post‐natal diagnosis was studied.

### 2.3 Clinical Data

All available clinical data were recorded including associated neurological and extra‐neurological malformations (e.g., cardiac and vascular anomalies, skin abnormalities, or renal malformations), neurosensorial disorders (visual or auditory impairments), epilepsy, and neurosurgical history (such as ventriculocisternostomy or shunting procedure).

Neurodevelopmental assessment included psychomotor development milestones (motor and language acquisition), and for school‐aged patients, the presence or absence of learning disabilities (including attention‐deficit/hyperactivity disorder and oral and/or written language impairments) or intellectual disability. Rehabilitation needs and cognitive assessment results, such as Wechsler scales, were also specified when available.

Patients' development was classified into four categories: (i) typical development (no developmental delay and normal school level); (ii) learning disability with normal intellectual skills (language delay, attention deficit, coordination development disorder, and school aid); (iii) severe neurodevelopmental disorder (no walking and/or intellectual disability); and (iv) death.

Foetal and postnatal genetic analyses were also documented when available, specifying the type of tests performed, including karyotype, chromosomal microarray analysis (CMA), Genome sequencing (GS), and targeted gene panels. Only pathogenic or likely pathogenic variants were mentioned.

Patients with DWM or BPC were classified into two groups: ‘Associated’ forms (patients with additional clinical malformations and/or genetic disorder and/or hydrocephalus requiring surgery) and ‘Isolated’ forms.

### 2.4 Statistics

Only data from the BPC and DWM groups were analysed. We used descriptive statistics to compare anatomical and neurodevelopmental data within the two groups or to explore other variables (isolated vs. associated, hydrocephalus or TVA value). Results are presented as median and interquartile range, or mean and standard deviation, as appropriate. Categorical variables were summarized as numbers and percentages. Analyses and graphs were performed using R Statistical software V4.2 [16].

### 2.5 Ethics

The study was registered at the local Research department of the CHU of Lyon (IRB 00013204, AGORA n°24‐5348). Clinical data were documented during routine clinical follow‐up; neither photographs nor blood samples were obtained as part of this study. In accordance with studies not directly involving patients, no further written consent was required from the parents [17].

## 3 Results

### 3.1 Population

In total, 35 foetal MRI scans were included between 2006 and 2021. Seven foetuses were excluded due to medical termination of pregnancy and one foetus was lost to follow‐up. Twenty‐seven patients met the inclusion criteria. Prenatal MRIs were performed at a median gestational age of 31 weeks of gestation (20–38 weeks).

### 3.2 Imaging Data

**FIGURE 2:**
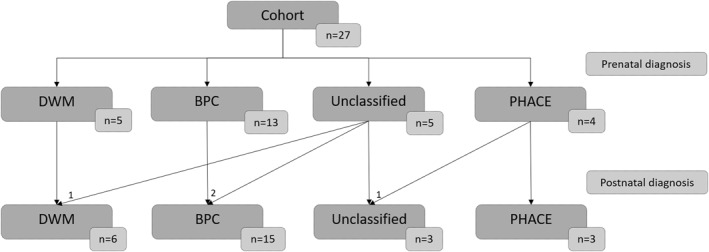
Foetal and postnatal distributions of the different groups. DWM: Dandy Walker malformation; BPC: Blake's pouch cyst. Among the three postnatally unclassified patients, patient TRS13 was “in between” DWM and BPC, in contrast to patients L6 and L8 who did not fit any diagnosis.

Four groups of patients could be identified in accordance with postnatal imaging (Figure 2): DWM, BPC, PHACE syndrome and unclassified patients.

**FIGURE 3:**
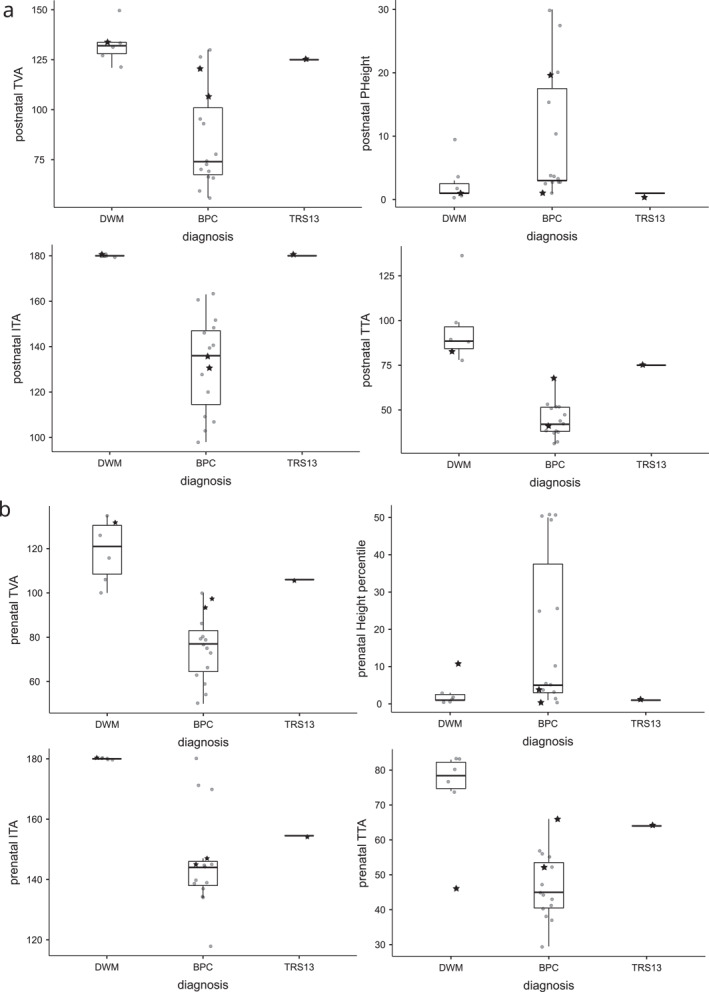
(a) Individual *postnatal* variables according to three groups: DWM (*N* = 6), BPC (*N* = 15) and unclassified (*N* = 1; TRS13). Height: vermian height; ITA: intra‐tentorial angle; TVA: tegmentovermian angle; TTA: tegmentotentorial angle. The stars refer to the four unclassified foetuses (Figure S2). (b) Individual *foetal* variables according to three groups (previously defined according to postnatal imaging): DWM (*N* = 6, including unclassified foetus TRS7 [star]), BPC (*N* = 15, including unclassified foetuses L4 and TRS12 [stars]) and patient TRS13, unclassified on postnatal MRI.

**FIGURE 4:**
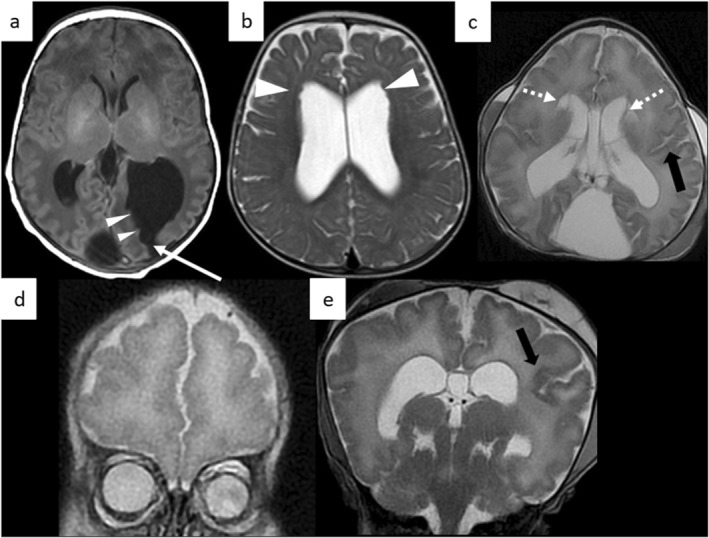
Postnatal MRI showing associated cerebral malformations. (a) Patient L13 (BPC). T1‐weighted axial image showing left occipital schizencephaly (white arrow) and subependymal nodular heterotopia (arrowheads) along the enlarged left occipital horn. (b) Patient TRS13 (unclassified DWM or BPC). T2‐weighted axial slice showing enlarged lateral ventricles with bilateral frontal subependymal nodular heterotopia (arrowheads). (c–e) Patient TRS14 (DWM). T2‐weighted axial (c) and coronal (d, e) planes showing bilateral frontal periventricular pseudocysts (dotted arrows) and left perisylvian polymicrogyria (black arrow) (right perisylvian polymicrogyria is not clearly visible on these slices). The olfactory bulbs and sulci were not visible in this patient.

- Six patients had a postnatal diagnosis of DWM (Figure 3a; Table 1; Figure S1), presenting with an overall upward orientation of the tentorium with an open TTA (over 78°, with a median of 88°) without any detectable intra‐tentorial change (ITA = 180°). In this group, the TVA was high, with a median of 132°.
- Fifteen patients had a diagnosis of BPC (Figure 3a, Table 1; Figure S1), presenting with a downward orientation of the anterior part of the tentorium with a sharp TTA (under 68°, median angle of 42°), and upward displacement of the distal part leading to an obvious ITA (< 180°). The TVA (with a median angle of 74°) was lower than that in the DWM group. The TTA values did not overlap between these two groups. A ‘grey zone’ was observed between the lower TTA of the DWM group (78°) and the higher TTA of the BPC group (68°). Additional malformations were observed in both groups (Figure 4a,c–e).
- Three patients fulfiled a diagnosis of PHACE syndrome on postnatal MRI, and did not require further measures for diagnosis. All were correctly recognized on foetal MRI. In accordance with the MRI diagnosis, all three neonates also had a facial haemangioma.
- Three patients could not be classified, even postnatally. Patient TRS13 had some criteria in favour of DWM and some in favour of BPC (Figures 3a and 4b, Figure S2b). His postnatal TTA (75°) was in the ‘grey zone’ mentioned above. Patients L6 and L8 had atypical MRI features (Figure S3(a–d)) that did not fit with any of the diagnostic criteria (either DWM or BPC). Foetal MRI of patient L8 suggested a PHACE syndrome, which was not confirmed postnatally.

Several foetal parameters were analysed to evaluate their relevance and reliability in predicting postnatal diagnosis of either DWM or BPC (Table 1 and Figure 3b). Vermian height was not discriminant and a wide overlap was observed between the two groups. More than a half of the BPC foetuses had a very low vermian height, less than the 10^th^ percentile. Vermian morphology was deemed impossible to determine in two thirds of the BPC foetuses. Distal change of the tentorium with an ITA < 180° was observed in all but one BPC foetus: TRS8, who was classified in the BPC group because of a clear downward orientation of the tentorium (TTA 55°). Three foetuses could not be classified prenatally: two BPC patients with a large TVA (TRS12 and L4; Figure 3b, Figure S2c,d) and one DWM patient (TRS7) with a sharp TTA (46°), overlapping with the BPC group (Figure 3b and Figure S2a). Interestingly, the TVA was a continuous variable with a common ‘cut‐off’ angle at around 100°. Ventriculomegaly was much more frequent in the DWM group but was still noted in a quarter of BPC patients.

**TABLE 1:** Imaging data of 21 patients, who could be classified in accordance with the classical description of either DWM or BPC.

	DWM (6 patients)		BPC (15 patients)		Relevance and reliability for diagnosis	
Imaging parameters	Foetal	Postnatal	Foetal	Postnatal	Foetal	Postnatal
ITA Med [min‐max]	180° (no angle)	180° (no angle)	144° [118°–180°]	136° [98°–163°]	Overlapping (one outsider)	Not overlapping
TTA Med [min‐max]	78° [46°–83°]	88° [78°–136°]	45° [29°–66°]	42° [31°–68°]	Overlapping (one outsider)	Not overlapping
TVA Med [min‐max]	121° [100°–137°]	132° [121°–150°]	74° [50°–100°]	74° [56°–130°]	4 postnatal overlapping Not discriminant	
Fluid space length Med [min‐max]	41 mm [19–55]	50 mm [43–58]	35 mm [23–43]	46 mm [36–81]	Not discriminant	
Vermis height (percentile)	1 [1–30]	1 [1–10]	5 [1–50]	3 [1–30]	Not discriminant	
Vermian morphology impossible to determine	100% (6 foetuses)	100% (6 patients)	67% (10 foetuses)	31% (5 patients)	Not discriminant	
Ventriculomegaly	66% (4 patients)	66% (4 patients)	13% (2 patients)	40% (6 patients)	Not discriminant	

### 3.3 Clinical Data

Since one aim of the study was to compare clinical outcomes of patients with either DWM or BPC, only clinical data concerning these 21 patients (+ unclassified patient TRS13) are described in Table 2.

**TABLE 2:** Radiological and clinical features and genetic analyses of the 22 patients with BPC or DWM.

Patient	Postnatal diagnosis	A. Associated I. Isolated	Malformation or clinical sign F. Foetal discovery P. Postnatal discovery	Genetic analysis: F. Foetal testing P. Postnatal testing	Outcome: 1. Typical devt 2. Learning disab 3. Severe disab 4. Death	Clinical course	Neurosurgery for hydraulic issues type and age
TRS14	DWM	A	F. Severe MCD. Heart defect: VSD Brain: Polymicrogyria, olfactive bulb agenesis	F. Karyotype, CMA P. gene panel: Likely pathogenic variant in ***DPH1*** NM_001383.4:c.374 T > C (p.Leu125Pro) (homozygote)	4	**Died** at 5 months old	N.A.
TRS7	DWM	A	Hydrocephalus	No data	4	**Died** before 6 months old.	Very large hydrocephalus (no surgery)
L1	DWM	A		F. Karyotype P. CMA: Arr **16p13.11**(14,968,855–16,292,235)x1 dn	2	7 y.o., walking 18 months old, coordination disorder, ADHD, regular school with aid, WISC V: VCI 86, RFI 82, VSI 78	VC and CPS at 1.5 y.o.
L3	DWM	A	P. Tricuspid insufficiency embryotoxon	F. Karyotype P. CMA: Arr [GRCh38] **6p25.3p25.2**(163,032_3,415,822)x1 dn	2	14 y.o., walking 35 months, language delay, coordination troubles, high school with aid, WISC 5 VCI 92, FRI 106, VSI 94	VC, Cystostomy and VPS at 11 days old
L2	DWM	A		F. Karyotype P. Karyotype + CMA	2	15 y.o., walking 15 months old, language troubles, coordination troubles, ADHD, regular school with aid, WISC‐5: VCI 85, RFI 106, VSI 89	VC and CPS at 1 months old
TRS15	DWM	A	P. Claude Bernard Horner, left deafness Patent foramen ovale	F. Karyotype, CMA P. GS: no pathogenic variant	1	3 y.o., walking 15 months old, mainstream development	/
TRS13	Unclassified DWM or BPC	A	F. Heart defect: VSD F. Subependymal heterotopias	P. Karyotype + CMA	1	7 y.o., walking 12 months old, typical development, regular schooling, WPPSI‐4: VCI 95, FRI 86, VSI 82	/
TRS3	BPC	A	P. Heart defect: VSD	F. Karyotype + CMA	2	5 y.o., walking 15 months old, coordination developmental disorder, normal schooling	VPS at 7 days old
TRS2	BPC	A	P. Noonan Sd: growth delay, exophthalmia, pulmonary valve stenosis	F. Karyotype, CMA P. Gene panel, pathogenic variant in ***KRAS*** NM_004985.5: c.178 G > A (p.Gly60Ser) (de novo)	2	3 y.o., walking 21 months old; language delay, ADHD, autistic features, adapted schooling, WPPSI‐4: FRI 99, VSI 79	VPS at 3 months old
L13	BPC	A	F. Schizencephaly and subependymal heterotopias	No genetic analyses	2	5 y.o., walking 24 months old, language delay, ADHD, preschool with aid	VC, DPS at 1 y.o.
L4	BPC	A		F. Karyotype P. Karyotype + CMA	2	13 y.o., walking 13 months old, language delay, coordination troubles, assisted schooling, WISC‐5: VCI 95, FRI 109, VSI 85	VPS at 15 months old
TRS1	BPC	A		P. Karyotype	1	3.5 y.o. Walking 12 months Typical development, regular schooling	CPS at 2 months old
TRS5	BPC	A		F. Karyotype + CMA	1	5 y.o., walking 12 months. Typical development, regular schooling	VPS (age not known)
TRS8	BPC	A	F. growth restriction, tethered spinal cord P. **Turner** Sd (horseshoe kidney)	F. Karyotype and CMA: **45X**	1	3 y.o., walking 18 months old, no language delay. Too young for school.	/
TRS12	BPC	I		F. Karyotype P. CMA	1	7 y.o., walking 14 months Typical development, regular schooling	/
TRS9	BPC	I		No genetic analysis	1	7 y.o. Walking 15 months Typical development, regular schooling	/
TRS10	BPC	I		No genetic analysis	1	7 y.o. Walking 12 months Typical development, regular schooling, WISC‐4: VCI 90 VTI 100, PRI 124	/
L12	BPC	I		No genetic analysis	2	15 y.o., walking 17 months, language delay, major anxiety, no schooling due to psychiatric disorders. Normal intellectual skills.	/
TRS4	BPC	I		F. Karyotype + CMA	1	24 months old. Walking 13 months Mainstream development	/
TRS 18	BPC	I		F. CMA	1	2 y.o., walking before 18 months	/
TRS11	BPC	I		No genetic analysis	1	3 y.o., walking 15 months old no delay	/
TRS6	BPC	I		No genetic analysis	1	6 y.o., walking 15 months old typical development, regular schooling	/

Abbreviations: ADHD: Attention Deficit Hyperactivity Disorder; BPC: Blake pouch's cyst; CMA (chromosomal microarray); CPS: cystoperitoneal shunt; DWM: Dandy Walker malformation; FRI: Fluid Reasoning Index; GS: Genome Sequencing; MCD: Multiple Congenital Defect; PRI: Perceptive Reasoning Index; Sd: syndrome; STI: Speed treatment index; VC: Ventriculo‐cisternostomy; VCI: Verbal Comprehension Index; VPS: ventriculoperitoneal shunt; VSD: Ventricular Septal Defect; VSI: Visual Spatial Index; WISC: Wechsler Intelligence Scale for Children; WPPSI: Weschler Preschool and Primary Scale of Intelligence; y.o.: years old.

In the DWM group, all six patients had additional malformations and/or microdeletion and/or major hydrocephalus. Two died before 6 months of age. The four surviving patients could walk at a mean age of 20 months. Three patients had hydrocephalus requiring neurosurgery and learning disabilities with intellectual skills in the normal range. No patient had an intellectual disability. One patient with unilateral deafness had normal school level and no surgery.

In the BPC group, seven out of 15 patients (47%) had an ‘associated’ form with additional clinical and/or genetic disorders and/or hydrocephalus requiring neurosurgery (six patients). Eight patients had an apparently ‘isolated’ form with normal genetic testing, performed on only three of them. The mean follow‐up was 5 years (2–15 years). Walking was acquired at an average age of 15,6 months (12–24 months), a little later in the ‘associated’ BPC group (mean: 16.4 months) than in the ‘isolated’ group (mean: 14.9 months). Ten out of 15 patients (66%) had typical development with normal school level. Five patients (33%) had learning disabilities (with preserved intellectual abilities): four with hydrocephalus requiring neurosurgery (three of them with additional malformation) and one with apparently isolated BPC but without genetic testing available.

**FIGURE 5:**
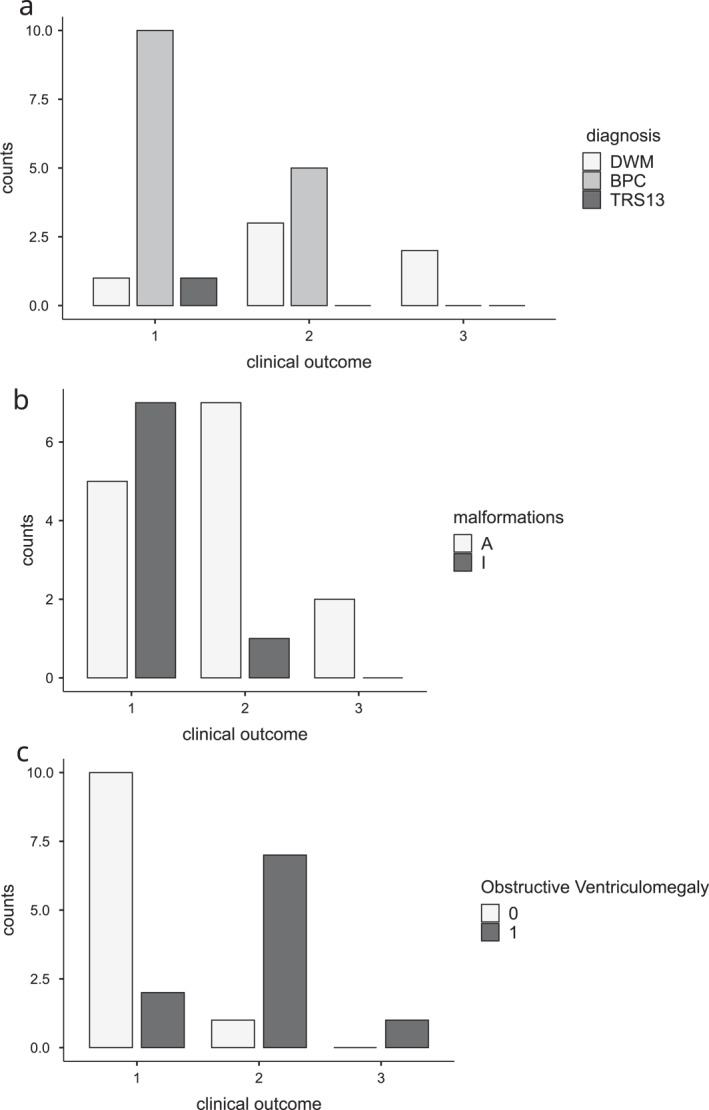
Development according to three different clinical parameters. (a) Diagnosis: DWM, BPC, or unclassified (*N* = 22). (b) Associated (A) versus isolated (I) (*N* = 22). (c) Hydrocephalus requiring surgery (H+) versus no hydrocephalus (H−) (*N* = 21). Development was classified into three categories: typical development (1), learning disability with preserved intellectual skills (2), and death (3).

In summary, patients from the DWM group had a worse outcome than those from the BCP group. Nevertheless, a third of the BPC patients also had learning disability, most of them with additional clinical features (Table 2; Figure 5a). No patient had seizures, even those with subependymal heterotopias and schizencephaly.

Regarding ‘associated’ versus ‘isolated’ CMPF (pooling the 22 patients with either DWM or BPC, + unclassified patient TRS13), the development was less favourable for patients with ‘associated’ CMPF compared to isolated forms (Figure 5b). In the ‘associated’ group (14 children), there were two deaths, five with learning disabilities (with preserved intellectual skills), and only five with typical development (36%), compared to the ‘isolated’ group (8 children) in which only one had learning disability and seven had typical development (87%).

Looking more specifically at the impact of hydrocephalus, among 21 patients (excluding one patient with DWM and multiple congenital defects who died), 10 (48%) had hydraulic complications, nine required neurosurgery, and one died in the context of very severe hydrocephalus, for which neurosurgical intervention was not considered due to disease severity. Seven had learning disabilities and only two showed typical development (20%). By contrast, among 11 patients who did not require surgery, one had learning disabilities and 10 (91%) had typical development (Figure 5c).

**FIGURE 6:**
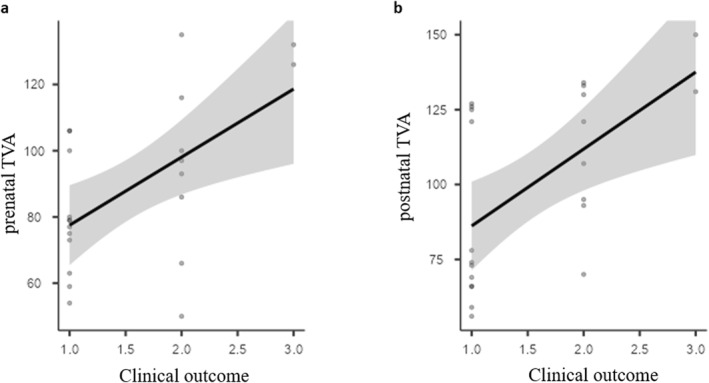
Correlation matrix (Pearson test) between TVA and clinical outcome classified into three categories: typical development (1), learning disability (2), and death (3). (a) Correlation between *foetal* TVA and clinical outcome (correlation coefficient = 0.561, *p* = 0.006). (b) Correlation between *postnatal* TVA and clinical outcome (correlation coefficient = 0.568, *p* = 0.006).

Since the TVA is an indirect marker of the volume of retrocerebellar fluid space, we also assessed its prognostic value. The median TVA value in patients with obstructive hydrocephalus appears to be higher than that in the group without hydrocephalus (Figure S4b). The two patients who died early had a *foetal* TVA above 120°; conversely, all patients with typical development had a *foetal* TVA below 110° (Figure 6a; Figure S4a). Despite widespread distribution of the TVA within the subgroups with learning disabilities, significant correlations were noticed between foetal TVA and clinical outcome (Figure 6a) as well as between postnatal TVA and clinical outcome (Figure 6b).

## 4 Discussion

Among cystic malformations of the posterior fossa, classification between BPCs and DWM remains confusing; thus, anatomical criteria should be defined more precisely in order to differentiate between these entities, especially for foetuses with a large opening of the fourth ventricle, as this represents a critical issue for prenatal counselling. In the literature, no series including DWM and large Blake's pouches (TVA > 40°) have addressed this issue.

In this study, despite a double‐blind investigation of pre‐ and post‐natal MRI by two experts in foetal brain imaging, it was not possible to classify 22% (6/27) of foetuses using the classic diagnostic criteria for DWM, BPC and PHACE syndrome. A positive correlation for foetal/postnatal imaging of 78% (21/27 of patients) may be considered a good result; however, in the frame of prenatal diagnosis, the inability to differentiate between DWM and BPC is a matter of concern when considering the possible stark difference in prognosis between these entities.

The three PHACE syndromes were accurately diagnosed using the ‘tilted telephone receiver sign’ [15], corroborated by postnatal abnormalities including facial haemangioma and vascular defects. Nevertheless, frank asymmetry between the two hemispheres may mimic this radiological sign, such as in patient L8 of this series, leading to a misdiagnosis of PHACE syndrome on foetal MRI.

Concerning the diagnosis of DWM and BPC, as already published [3], the most relevant diagnostic criteria for differentiating between DWM and a large BPC is the overall upward displacement of the tentorium in DWM with an open TTA, compared to the downward orientation of the proximal part of the tentorium (sharp TTA) interrupted by upward displacement of the distal part leading to an obvious ITA (< 180°) in BPC. Using the combination of these two angles led to a postnatal diagnosis in 21/22 patients with a cut‐off TTA between 68° and 78°. Interestingly, the patient TRS13, who could not be included in either of these two distinct groups, had a TTA of 75°, in the ‘grey zone’.

Foetal TTA was higher for DWM (min: 46°, max: 83° with a median of 78°) than for BPC (min: 29°, max: 66°, median angle of 45°), except for one DWM foetus. An ITA < 180° was observed in all but one BPC foetus. For one foetus, the choice of two different sagittal planes led to two different diagnoses, thus highlighting the difficulty in selecting an appropriate plane in prenatal settings. Indeed, although TTA and ITA can be considered fairly good parameters, anatomical pitfalls cannot be totally avoided, since three foetuses with either DWM or a large BPC could not be correctly classified on foetal MRI, showing the limits of these parameters in prenatal settings. Vermian height, retrocerebellar lake size, and vermis morphology have historically been the criteria used to distinguish DWM from other malformations [11, 12]. However, in this cohort, these variables did not discriminate between DWM and BPC, either prenatally or postnatally, as already pointed out [3, 11]. TVA has also been described in the literature [12, 18] as a useful tool to differentiate between DWM and BPC. In this study, the median postnatal TVA was higher for DWM (132°) compared to BPC (74°), but extreme values overlapped between the two groups and this parameter was not discriminant for three large BPC patients with a postnatal TVA above 120°.

The foetal TVA cut‐off (at around 100°) observed in our series is much higher than the previous cut‐off described by Volpe et al. [19] with a foetal TVA < 30° for BPC and > 45° for DWM. Two reasons might explain this apparent discrepancy. First, our inclusion criterion was TVA > 40°, which identified DWM and large BPC. Second, the median gestational age at MRI in our cohorts (31 weeks) was later than the median (21–30 weeks) in Volpe et al. [19]. Nevertheless, the lack of BPC patients with an angle above 30° in the series of Volpe et al. is surprising. Some BPC patients might have been classified as DWM on the basis of a ‘large cyst’ without further criteria (TTA and ITA). Similarly, Boddaert et al. 2003 [8] described typical neurodevelopmental outcome in 82% of patients with ‘DWM’ which is unusual compared to what has been reported in other series [7]. We speculate that some were misdiagnosed as large BPC, since these authors observed normal vermian lobulation [8], which is a classic diagnostic criterion for BPC; moreover, one of the MR images in these reports fulfils our criteria for a large BPC.

This study focussed on the use of MRI criteria to distinguish DWM from BPC. As mentioned, all patients were referred due to CMPF identified during prenatal ultrasound scanning. In our experience, when ultrasound can be performed using the posterior fontanel approach, the different measures discussed in our study are also available. However, the purpose of our study was not to evaluate the accuracy of ultrasound in this field.

Conventionally, distinct embryological pathways are reported to lead to BPC and DWM. Persistent Blake's pouch is thought to result from failure of fenestration of the foramina of Lushka and Magendie [20]. The anterior membranous area is defective in DWM while it is intact in BPC [21]. Moreover, it has been suggested that DWM results from an early insult in the cerebellar rhombic lip [22]. Despite these embryological considerations, our study might suggest a continuum between DWM and large BPC.

As it may not always be possible to distinguish between DWM and large BPC, we looked for other relevant prognostic factors. Regarding ‘associated’ versus isolated CMPF, development was clearly less favourable in the group with associated malformations or genetic diagnosis, as already published [4, 8]. However, it should be noted that so called ‘associated’ CMPF is a very heterogeneous group, and some genetic findings such as Turner syndrome do not affect neurodevelopment. Conversely, genetic mutations in specific genes, such as *ZIC1‐ZIC4, SETD2* and *FOXC1* [23, 24]_,_ are known to be associated with poor prognosis. Interestingly, in our series one patient (L3, DWM) with a 6p25 deletion [25] involving *FOXC1* had preserved intellectual skills. In addition, four genetic diagnoses were not previously described to be associated with DWM (del 16p13.11, including *NDE1* [26] and *DPH1* pathogenic variant [27]) or BPC (Noonan and Turner syndromes). In the study of Volpe et al. [28], two of 27 BPC patients had genetic defects (1q21 deletion and trisomy 21 mosaicism). In order to better define prognosis, systematic foetal CMA and exome sequencing may be proposed to all pregnant women faced with a diagnosis of large CMPF, as already proposed for cases of agenesis of the corpus callosum, providing parents with reliable prenatal advice [29].

Another prognostic factor to take into account is the hydraulic issue associated with enlarged retrocerebellar fluid space. A typical development was observed in only 20% of patients with hydrocephalus compared with 91% in patients who did not have hydrocephalus. As expected, patients with a high TVA underwent surgery more frequently than those with a low TVA, and a significant correlation between foetal TVA and clinical outcome was observed. Further studies are needed to confirm whether TVA is a reliable prognostic factor, since it is an easily measured parameter.

## 5 Conclusion

Inaccuracies persist in the prenatal imaging diagnosis and prognosis of CMPF with a marked opening of the V4 (notably DWM and large BPC). In this study, the most relevant diagnostic criteria for differentiating between DWM and large BPC was the overall upward displacement of the tentorium in DWM with an open TTA (> 78°), compared to the downward orientation of the proximal part of the tentorium (sharp TTA < 68°) in BPC, with upward displacement of the distal part leading to an obvious ITA. Nevertheless, our study suggests that there may be a continuum between the two entities. Beyond the pitfalls of nosographic issues, our study confirms that ‘associated’ malformations (either DWM or BPC) correlate with a worse prognosis. Thorough foetal scanning and extended genetic analyses may be useful for prenatal counselling. In addition, although TVA is not a good parameter for differential diagnosis between DWM and BPC, it may be a promising prognostic factor, as an indirect marker of fluid space volume, which correlates with surgical requirement and developmental outcome.

## Funding

The authors have nothing to report.

## Conflicts of Interest

The authors declare no conflicts of interest.

## Supporting information


**Figure S1a:** Pre and post‐natal sagittal MRI of 12 among 22 patients diagnosed with either DWM or BPC (including patient TRS13, unclassified). Patients are shown according to their post‐natal TVA angle, in decreasing order. Upper row: prenatal; lower row: post‐natal.

**Figure S1b:** Pre and post‐natal sagittal MRI of 10 among 22 patients diagnosed with either DWM or BPC (including patient TRS13, unclassified). Patients are shown according to their post‐natal TVA angle, in decreasing order. Upper row: prenatal; lower row: post‐natal.

**Figure S2:** MRI of four unclassified *foetuses* shown on sagittal, coronal and axial planes (T2‐weighted images) (a‐d). The upper row illustrates the prenatal appearance of each patient and the lower row illustrates postnatal appearance. (a) *Patient TRS7 (postnatal DWM).* Postnatal MRI findings were in keeping with DWM, due to overall upward orientation of the tentorium with an open TTA (88°) and without any detectable ITA. On foetal MRI (32+2 weeks), high TVA and an absence of intra‐tentorial angle were suggestive of DWM, however, the overall downward orientation of the tentorium with a sharp TTA (46°) suggested a large BPC. Therefore, the foetus was deemed unclassified. (b) *Patient TRS13 (unclassified postnatally, DWM or BPC).* On postnatal MRI, no detectable ITA was observed. The overall upward orientation of the tentorium suggested DWM. Moreover, the TVA was quite high (125°) and the morphology of the vermis was impossible to evaluate. Nevertheless, the TTA was 75°, a little too sharp to indicate DWM and the vermis did not appear to be small on sagittal plane. On foetal MRI (34 weeks), TVA (106°) was quite high for BPC but the tentorium was not markedly elevated, with a sharp TTA (112°) and a subtle elevation of the distal part of the tentorium (ITA = 154°), suggesting BPC. The foetus was unclassified. (c) *Patient TRS12 (postnatal BPC).* On postnatal MRI, BPC was confirmed with a downward orientation of the anterior part of the tentorium (sharp TTA, 68°) and an elevation of the distal part leading to an obvious intra‐tentorial angle (ITA = 140°). On foetal MRI (24 weeks), there was concern for an intra‐tentorial angle (ITA = 145°), suggesting BPC but it was difficult to precisely localize the proximal part of the tentorium. Moreover, the vermis appeared very small, the TVA (100°) was high, and the TTA (66°) was relatively open for a BPC. The foetus was unclassified. (d) *Patient L4 (postnatal BPC).* On postnatal MRI, BPC was confirmed with a downward orientation of the proximal part of the tentorium (sharp TTA of 42°), and an elevation of the distal part leading to an obvious intra‐tentorial angle (ITA: 131°). On foetal MRI, the TVA was quite high without a clear upward orientation of the tentorium. On midsagittal slice at the level of the Sylvian aqueduct, a sharp TTA (56°) was measured and a subtle intra‐tentorial angle was discussed depending on the selected slice (Supplementary Figure 1). The foetus was unclassified.

**Figure S3:** MRI of two unclassified patients (L6 and L8) shown on sagittal, coronal and axial planes (T2‐weighted images). (a) Patient L6: foetal MRI at 30 weeks of gestation. The proximal part of the tentorium shows a downward orientation excluding DWM but no ITA was clearly detectable. The abnormal morphology of the vermis, which appears to be incomplete, could suggest an ischaemic lesion, but a thin corpus callosum was also observed, which is more consistent with a dysgenetic process. (b) Patient L6: postnatal MRI (six days old). BPC could be discussed but the thin corpus callosum with partial agenesis, pons hypoplasia, dysgenetic and hypoplastic cerebellar hemispheres were atypical. (c) Patient L8: foetal MRI at 36 weeks. Asymmetric hemispheres and a ‘tilted telephone receiver’ appearance (coronal plane) suggested a PHACE syndrome. (d) Patient L8: postnatal MRI. The same findings were observed postnatally. However, no facial haemangioma was observed, which makes the diagnosis of PHACE syndrome questionable. The asymmetry of the two hemispheres and the brainstem hypoplasia were atypical for DWM.

**Figure S4:** Individual values of the tegmento‐vermian angle (TVA) according to several parameters on foetal (left) and postnatal (right) MRI. (a) TVA according to clinical outcome, classified into three categories: typical development (1), learning disability with preserved intellectual skills (2) and death (3). (b) TVA according to hydraulic complications: no hydrocephalus (0) *versus* hydrocephalus requiring surgery (1).

## Data Availability

The data that support the findings of this study are available from the corresponding author upon reasonable request.
