# Supplementary figures and images for: Prenatal Diagnosis and Neurodevelopmental Outcome of Children With Marked Opening of the Fourth Ventricle: Challenges and Pitfalls in MRI Diagnostic Criteria
Source: Prenat Diagn. 2026 Feb 20;46(3):315–28. doi: 10.1002/pd.70093 (PMC12978516; doi:10.1002/pd.70093)

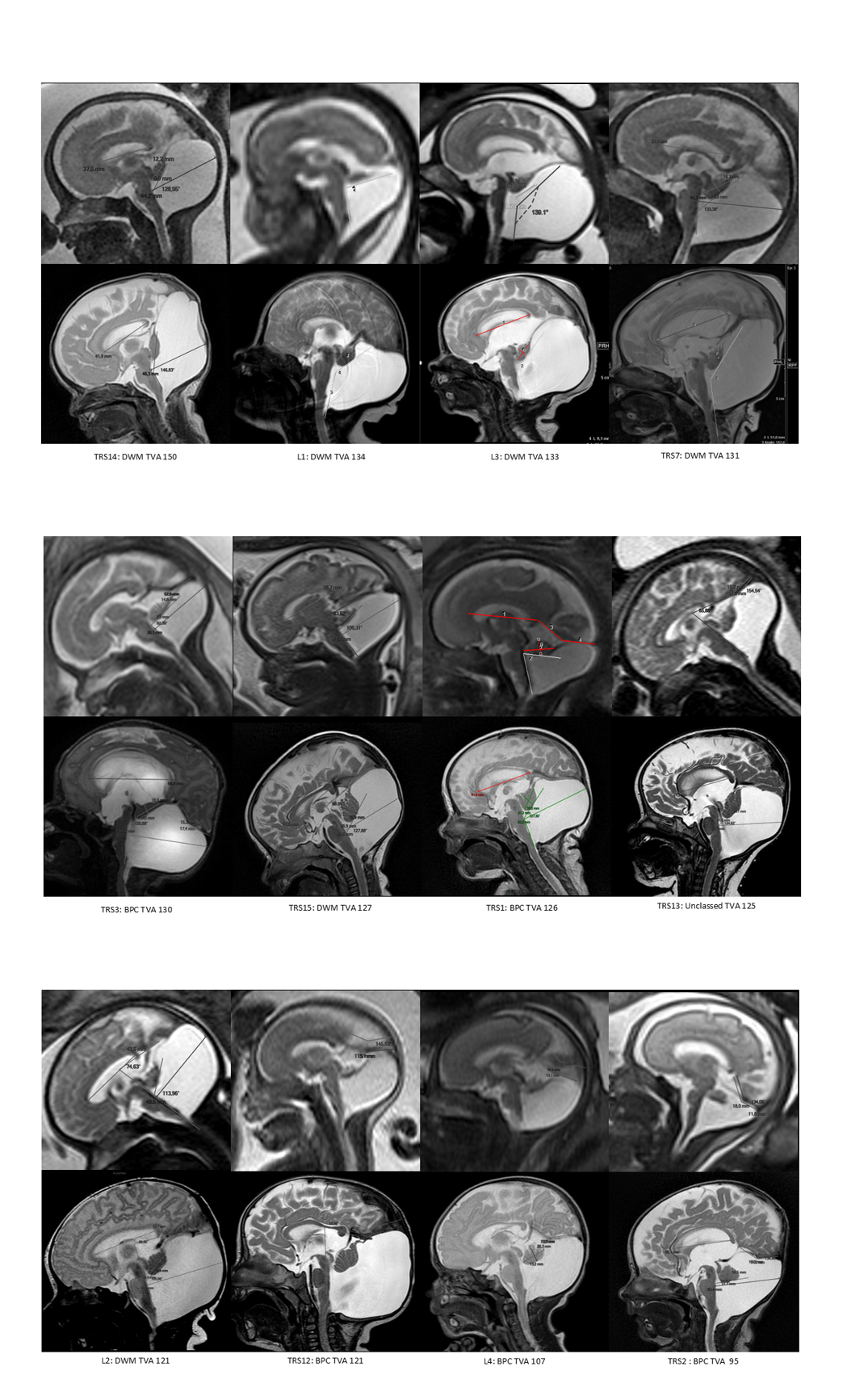

Supplement: Supplementary file 1 — Figure S1a: Pre and post‐natal sagittal MRI of 12 among 22 patients diagnosed with either DWM or BPC (including patient TRS13, unclassified). Patients are shown according to their post‐natal TVA angle, in decreasing order. Upper row: prenatal; lower row: post‐natal. [file PD-46-315-s005.tif]

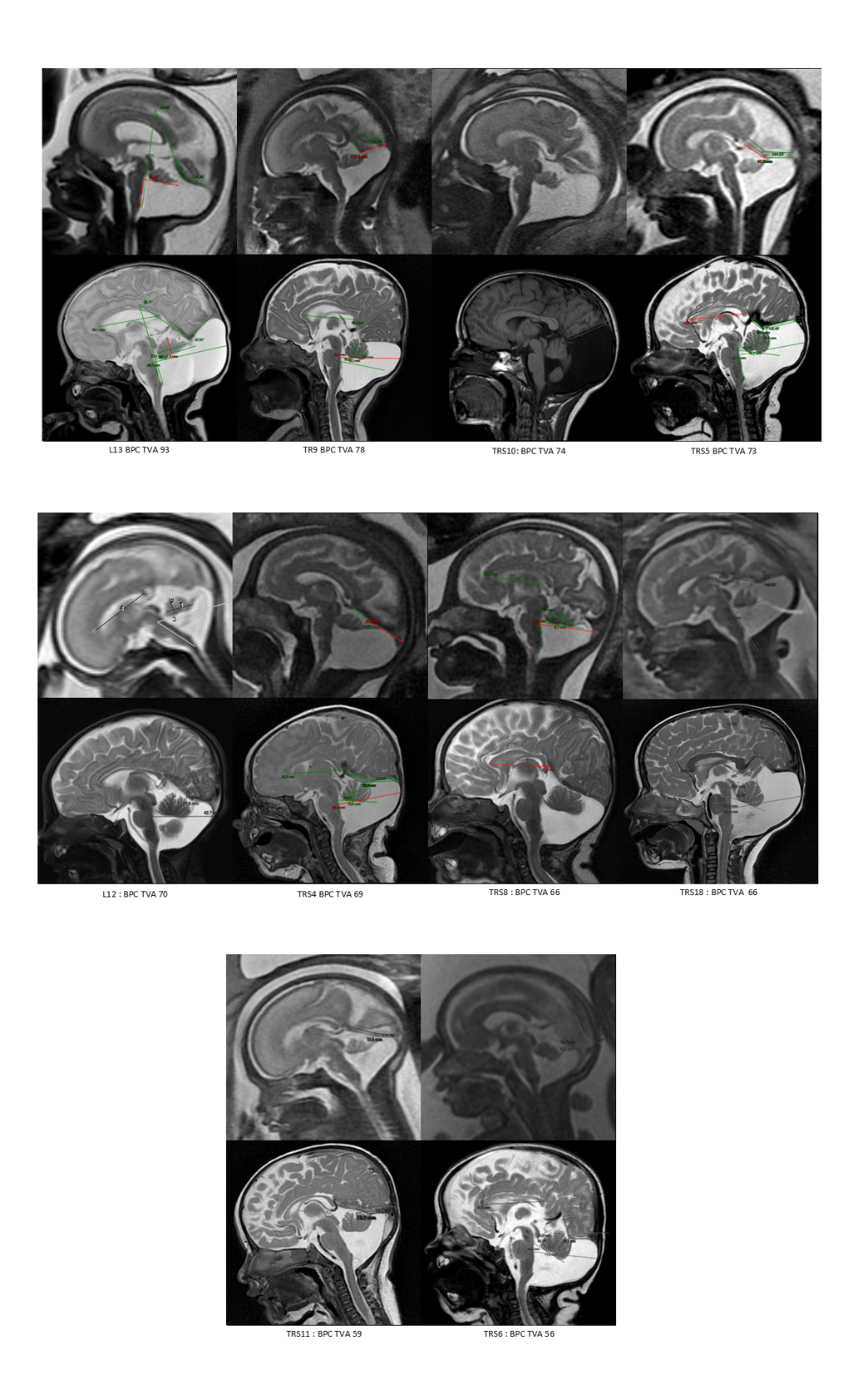

Supplement: Supplementary file 2 — Figure S1b: Pre and post‐natal sagittal MRI of 10 among 22 patients diagnosed with either DWM or BPC (including patient TRS13, unclassified). Patients are shown according to their post‐natal TVA angle, in decreasing order. Upper row: prenatal; lower row: post‐natal. [file PD-46-315-s003.tif]

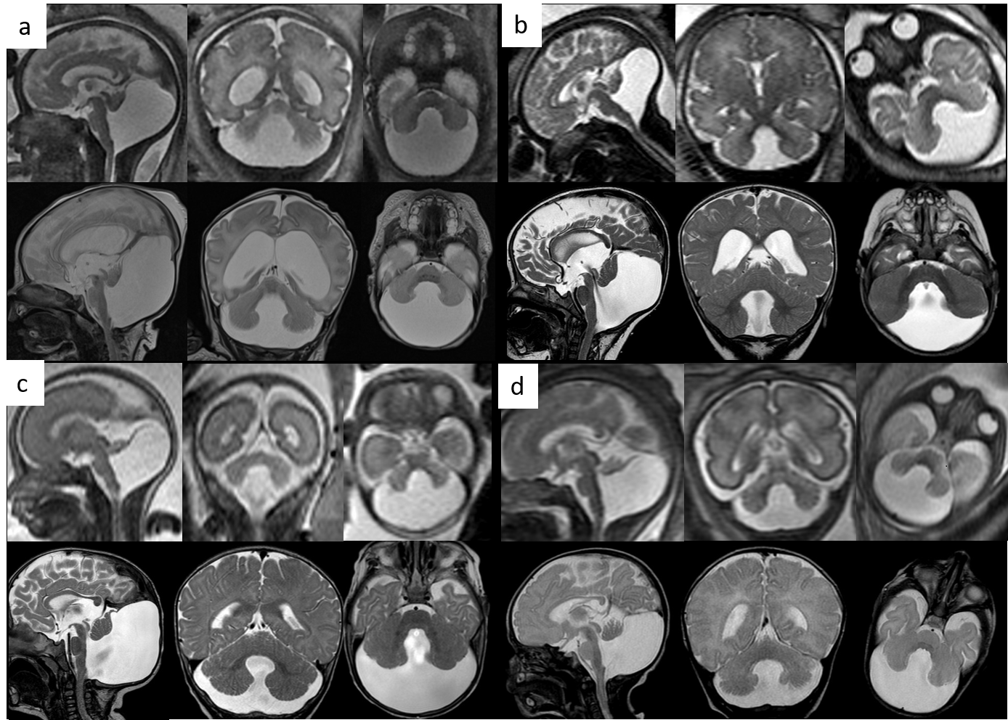

Supplement: Supplementary file 3 — Figure S2: MRI of four unclassified foetuses shown on sagittal, coronal and axial planes (T2‐weighted images) (a‐d). The upper row illustrates the prenatal appearance of each patient and the lower row illustrates postnatal appearance. (a) Patient TRS7 (postnatal DWM). Postnatal MRI findings were in keeping with DWM, due to overall upward orientation of the tentorium with an open TTA (88°) and without any detectable ITA. On foetal MRI (32+2 weeks), high TVA and an absence of intra‐tentorial angle were suggestive of DWM, however, the overall downward orientation of the tentorium with a sharp TTA (46°) suggested a large BPC. Therefore, the foetus was deemed unclassified. (b) Patient TRS13 (unclassified postnatally, DWM or BPC). On postnatal MRI, no detectable ITA was observed. The overall upward orientation of the tentorium suggested DWM. Moreover, the TVA was quite high (125°) and the morphology of the vermis was impossible to evaluate. Nevertheless, the TTA was 75°, a little too sharp to indicate DWM and the vermis did not appear to be small on sagittal plane. On foetal MRI (34 weeks), TVA (106°) was quite high for BPC but the tentorium was not markedly elevated, with a sharp TTA (112°) and a subtle elevation of the distal part of the tentorium (ITA = 154°), suggesting BPC. The foetus was unclassified. (c) Patient TRS12 (postnatal BPC). On postnatal MRI, BPC was confirmed with a downward orientation of the anterior part of the tentorium (sharp TTA, 68°) and an elevation of the distal part leading to an obvious intra‐tentorial angle (ITA = 140°). On foetal MRI (24 weeks), there was concern for an intra‐tentorial angle (ITA = 145°), suggesting BPC but it was difficult to precisely localize the proximal part of the tentorium. Moreover, the vermis appeared very small, the TVA (100°) was high, and the TTA (66°) was relatively open for a BPC. The foetus was unclassified. (d) Patient L4 (postnatal BPC). On postnatal MRI, BPC was confirmed with a downward orientation of the [file PD-46-315-s001.tif]

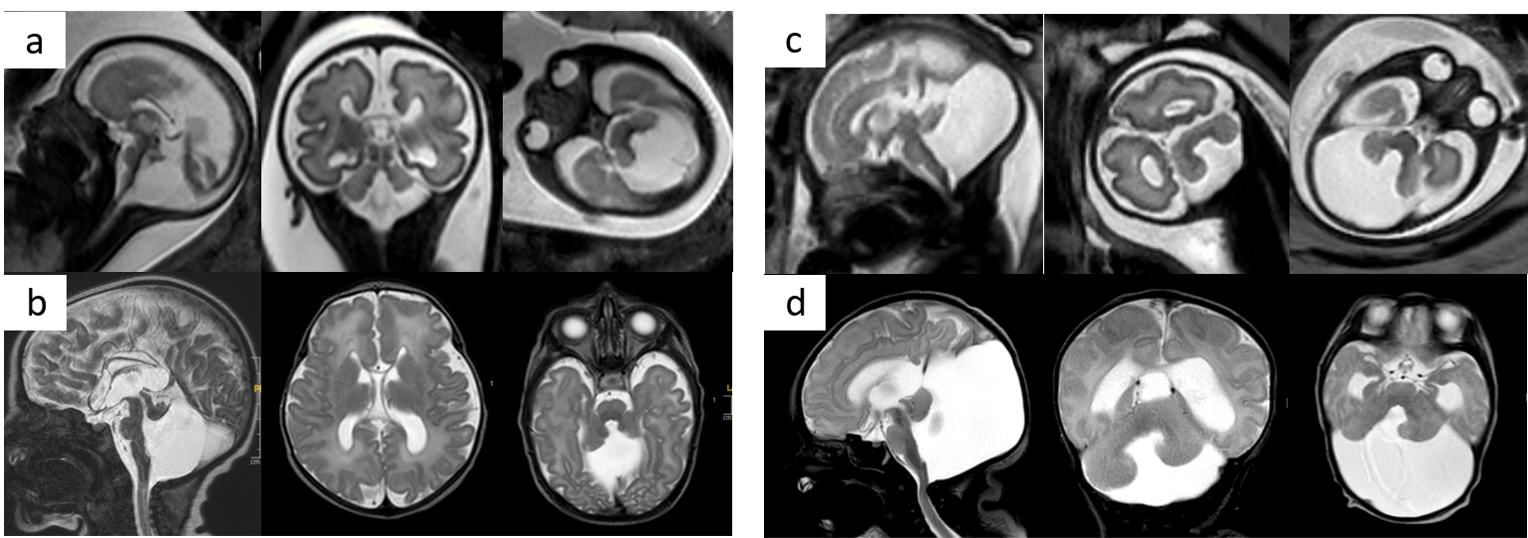

Supplement: Supplementary file 4 — Figure S3: MRI of two unclassified patients (L6 and L8) shown on sagittal, coronal and axial planes (T2‐weighted images). (a) Patient L6: foetal MRI at 30 weeks of gestation. The proximal part of the tentorium shows a downward orientation excluding DWM but no ITA was clearly detectable. The abnormal morphology of the vermis, which appears to be incomplete, could suggest an ischaemic lesion, but a thin corpus callosum was also observed, which is more consistent with a dysgenetic process. (b) Patient L6: postnatal MRI (six days old). BPC could be discussed but the thin corpus callosum with partial agenesis, pons hypoplasia, dysgenetic and hypoplastic cerebellar hemispheres were atypical. (c) Patient L8: foetal MRI at 36 weeks. Asymmetric hemispheres and a ‘tilted telephone receiver’ appearance (coronal plane) suggested a PHACE syndrome. (d) Patient L8: postnatal MRI. The same findings were observed postnatally. However, no facial haemangioma was observed, which makes the diagnosis of PHACE syndrome questionable. The asymmetry of the two hemispheres and the brainstem hypoplasia were atypical for DWM. [file PD-46-315-s004.tif]

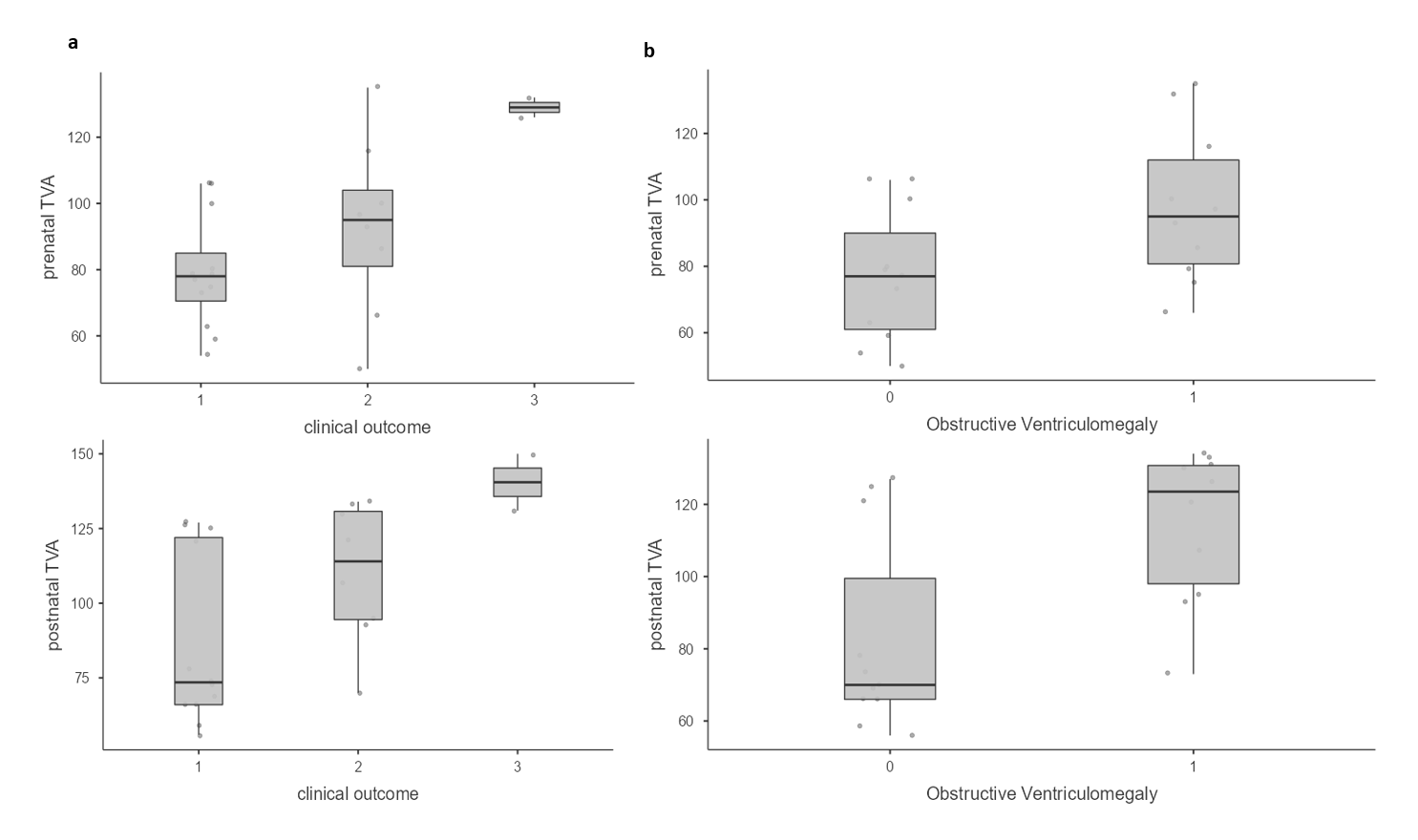

Supplement: Supplementary file 5 — Figure S4: Individual values of the tegmento‐vermian angle (TVA) according to several parameters on foetal (left) and postnatal (right) MRI. (a) TVA according to clinical outcome, classified into three categories: typical development (1), learning disability with preserved intellectual skills (2) and death (3). (b) TVA according to hydraulic complications: no hydrocephalus (0) versus hydrocephalus requiring surgery (1). [file PD-46-315-s002.tif]
